# Mitochondrial Respiration in Response to Iron Deficiency Anemia: Comparison of Peripheral Blood Mononuclear Cells and Liver

**DOI:** 10.3390/metabo12030270

**Published:** 2022-03-21

**Authors:** Christine Fischer, Lara Valente de Souza, Timea Komlódi, Luiz F. Garcia-Souza, Chiara Volani, Piotr Tymoszuk, Egon Demetz, Markus Seifert, Kristina Auer, Richard Hilbe, Natascha Brigo, Verena Petzer, Malte Asshoff, Erich Gnaiger, Günter Weiss

**Affiliations:** 1 Department of Internal Medicine II, Medical University of Innsbruck, Anichstrasse 35, 6020 Innsbruck, Austria; christine.fischer@i-med.ac.at (C.F.); lara.valente@i-med.ac.at (L.V.d.S.); chiara.volani@eurac.edu (C.V.); piotr.s.tymoszuk@gmail.com (P.T.); egon.demetz@i-med.ac.at (E.D.); markus.seifert@i-med.ac.at (M.S.); kristina.auer@labordiagnostik.tirol (K.A.); richard.hilbe@tirol-kliniken.at (R.H.); natascha.brigo@i-med.ac.at (N.B.); verena.petzer@i-med.ac.at (V.P.); malte.asshoff@i-med.ac.at (M.A.); 2 Christian Doppler Laboratory for Iron Metabolism and Anemia Research, Medical University of Innsbruck, Anichstrasse 35, 6020 Innsbruck, Austria; 3 Oroboros Instruments, Schöpfstrasse 18, 6020 Innsbruck, Austria; komlodi.timea@med.semmelweis-univ.hu (T.K.); luiz.garcia@oroboros.at (L.F.G.-S.); erich.gnaiger@oroboros.at (E.G.)

**Keywords:** anemia, iron deficiency, peripheral blood mononuclear cells, liver, mitochondrial function, OXPHOS, mitochondrial respiration, surrogate

## Abstract

Iron is an essential component for metabolic processes, including oxygen transport within hemoglobin, tricarboxylic acid (TCA) cycle activity, and mitochondrial energy transformation. Iron deficiency can thus lead to metabolic dysfunction and eventually result in iron deficiency anemia (IDA), which affects approximately 1.5 billion people worldwide. Using a rat model of IDA induced by phlebotomy, we studied the effects of IDA on mitochondrial respiration in peripheral blood mononuclear cells (PBMCs) and the liver. Furthermore, we evaluated whether the mitochondrial function evaluated by high-resolution respirometry in PBMCs reflects corresponding alterations in the liver. Surprisingly, mitochondrial respiratory capacity was increased in PBMCs from rats with IDA compared to the controls. In contrast, mitochondrial respiration remained unaffected in livers from IDA rats. Of note, citrate synthase activity indicated an increased mitochondrial density in PBMCs, whereas it remained unchanged in the liver, partly explaining the different responses of mitochondrial respiration in PBMCs and the liver. Taken together, these results indicate that mitochondrial function determined in PBMCs cannot serve as a valid surrogate for respiration in the liver. Metabolic adaptions to iron deficiency resulted in different metabolic reprogramming in the blood cells and liver tissue.

## 1. Introduction

Anemia is a global burden affecting more than 1.5 billion people worldwide [1,2,3]. The most prevalent form of anemia is iron deficiency anemia (IDA), mostly occurring in preschool children and women in reproductive age [1,2]. The main symptoms include fatigue, weakness, and pale skin along with reduced cardiovascular performance. Moreover, IDA may negatively affect the growth and cognitive development of children [1,4,5]. IDA is characterized by absolute iron deficiency caused by chronic blood loss, insufficient dietary intake, or a combination of both [1,5].

Iron is an essential trace element for life, as it is necessary for hemoglobin biosynthesis and for key metabolic enzymes involved in DNA replication, hormone synthesis, and mitochondrial bioenergetics [6,7]. Especially in mitochondria, iron is needed for heme synthesis, iron sulfur (Fe-S) cluster formation, and oxidative phosphorylation [8,9,10,11,12]. In the electron transfer system (ETS) complexes CI, CII, and CIII possess Fe-S clusters which are crucial for the synthesis of adenosine triphosphate (ATP).

Iron metabolism is tightly regulated by the liver-derived peptide hormone hepcidin, which mediates the degradation of ferroportin, the only cellular iron exporter known so far, thereby controlling iron absorption and iron recycling from macrophages [13]. Expression of hepcidin is controlled by multiple factors. Both iron deficiency and anemia reduce hepcidin expression, therefore increasing circulating iron levels and delivery to erythroid progenitor cells [3,13].

It has been shown previously that systemic iron deficiency and IDA reduce mitochondrial respiratory capacity in cardiomyocytes and skeletal muscle via the reduction of iron-rich mitochondrial electron transfer components and morphologic changes of mitochondria, such as reduced cristae structure [14,15,16,17,18]. In addition, mitochondria in hepatocytes of iron-deficient rats exhibit ultrastructural abnormalities, including an enlarged and rounded shape, therefore occupying an increased proportion of the cytoplasm due to their larger size, but not due to an increased number of mitochondria [19,20].

Despite the possible implications of these morphologic alterations on mitochondrial function, the impact of IDA on mitochondrial respiration in circulating peripheral blood mononuclear cells (PBMCs) and the liver has not been analyzed thus far. We studied these two systems, since the determination of mitochondrial function in PBMCs might represent an easily accessible surrogate reflecting mitochondrial functionality in the organs, such as the liver. Changes in the mitochondrial respiration of PBMCs have been observed in various diseases, including fatty liver disease, depression, or sepsis, and even in restless legs syndrome, where impaired mitochondrial function was linked to the indication of mitochondrial iron deficiency [21,22,23,24]. Therefore, the analysis of PBMCs could be an efficient and minimally invasive procedure when relating information from blood samples to organ function or disease state instead of collecting biopsies from the particular organ itself.

The aim of this study was a comparative analysis of mitochondrial respiration in PBMCs and the liver under steady-state control conditions and in the course of IDA.

## 2. Results

### 2.1. Effects of IDA on Hematological and Iron Parameters

**Figure 1:**
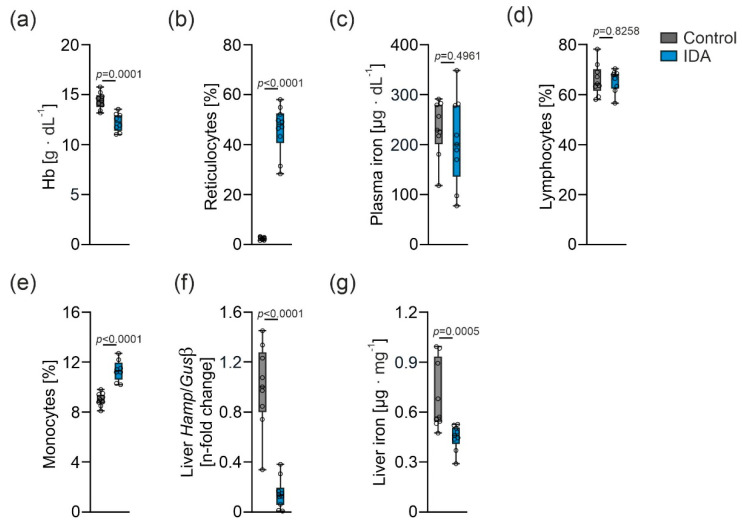
Alterations in blood and iron parameters caused by iron deficiency anemia (IDA). (**a**) Hemoglobin (Hb) concentration, (**b**) reticulocyte proportion, (**c**) plasma iron concentration, (**d**) lymphocyte fraction, (**e**) monocyte fraction, (**f**) liver *Hamp* mRNA expression relative to *glucuronidase beta* (*Gusβ*), and (**g**) liver iron content. Control: *N* = 9 rats, IDA *N* = 10 rats. An unpaired two-tailed Student’s *t*-test was applied for all normal distributed results; a Mann-Whitney U-test was applied for the nonparametric result shown in (**g**). Values are shown as median ± interquartile range. *p*-Values are shown in the graphs.

IDA was induced by phlebotomy for five consecutive days, as previously described [25]. As anticipated, the hemoglobin concentration was significantly lower in rats with IDA compared to the controls (Figure 1a). Moreover, the reticulocyte fraction was increased (Figure 1b). Plasma iron concentration did not differ significantly (Figure 1c). The lymphocyte fraction remained unaffected (Figure 1d), whereas an increased proportion of monocytes altered the PBMC composition in rats with IDA (Figure 1e). Hepatic mRNA expression (*Hamp*) of the master regulator of the iron metabolism, hepcidin, was decreased (Figure 1f). Along with that, the liver iron content was lower in the IDA animals compared to the controls (Figure 1g).

### 2.2. Mitochondrial Respiration Differs in PBMCs and Livers in Response to IDA

**Figure 2:**
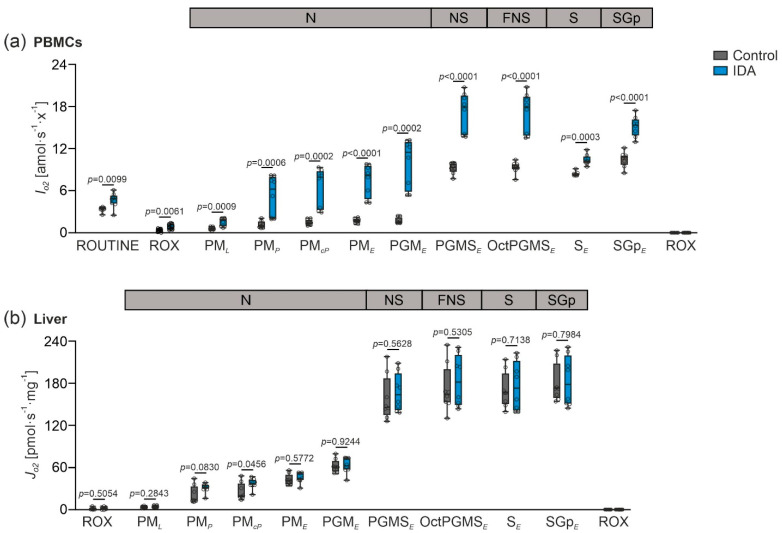
Effects of IDA on mitochondrial respiration in rat peripheral blood mononuclear cells (PBMCs) and liver. (**a**) Mitochondrial respiratory capacity in PBMCs with sequential titrations expressed as flow per cell (*I_O2_* [amol·s^−1^·x^−1^]); ROUTINE respiration measured in presence of isolated living PBMCs. Permeabilization of plasma membrane using digitonin: residual oxygen consumption (*Rox*, state ROX); pyruvate and malate (PM) in absence of adenosine diphosphate (ADP)-detecting NADH (N)-linked LEAK respiration (*L*), state PM*_L_*; kinetically saturating concentration of ADP-detecting OXPHOS capacity (*P*), state PM*_P_*; cytochrome *c* (c) for detection of mitochondrial outer membrane integrity, state PM*_cP_*; uncoupler titrations measuring electron transfer (ET) capacity (*E*), state PM*_E_*; glutamate (G) to measure N-linked ET capacity, state PGM*_E_*; succinate (S) to detect NS-linked ET capacity, state PGMS*_E_*; octanoylcarnitine (Oct) measuring FNS-linked ET capacity, state OctPGMS*_E_*; complex I inhibitor rotenone for detection of S-ET capacity, state S*_E_*; glycerol-3-phosphate (Gp) analyzing SGp-ET capacity, state SGp*_E_*; antimycin A measuring *Rox* (state ROX). (**b**) Mitochondrial respiratory capacity in the liver with sequential titrations expressed as flux per mass (*J_O2_* [pmol·s^−1^·mg^−1^]); *Rox* measured in the presence of liver homogenate; PM in the absence of ADP-detecting N-linked LEAK respiration, state PM*_L_*; kinetically saturating concentration of ADP-detecting OXPHOS capacity, state PM*_P_*; c for detection of mitochondrial outer membrane integrity, state PM*_cP_*; uncoupler titrations detecting ET capacity, state PM*_E_*; G to detect N-linked ET capacity, state PGM*_E_*; S to measure NS-linked ET capacity, state PGMS*_E_*; Oct to measure FNS-linked ET capacity, state OctPGMS*_E_*; rotenone to detect S-ET capacity, state S*_E_*; Gp to analyze SGp-ET capacity, state SGp*_E_*; antimycin A to measure *Rox* (state ROX). *N* = 8 rats per group. An unpaired two-tailed Student’s *t*-test was applied for all normal distributed states; a Mann-Whitney U-test was applied for the nonparametric states (**a**) PM*_P_*, PM*_cP_*, PGM*_E_*, S*_E_*; (**b**) ROX, PM*_P_*, SGp*_E_*. Values are shown as median ± interquartile range. *p*-Values are shown in the graphs.

**Figure 6:**
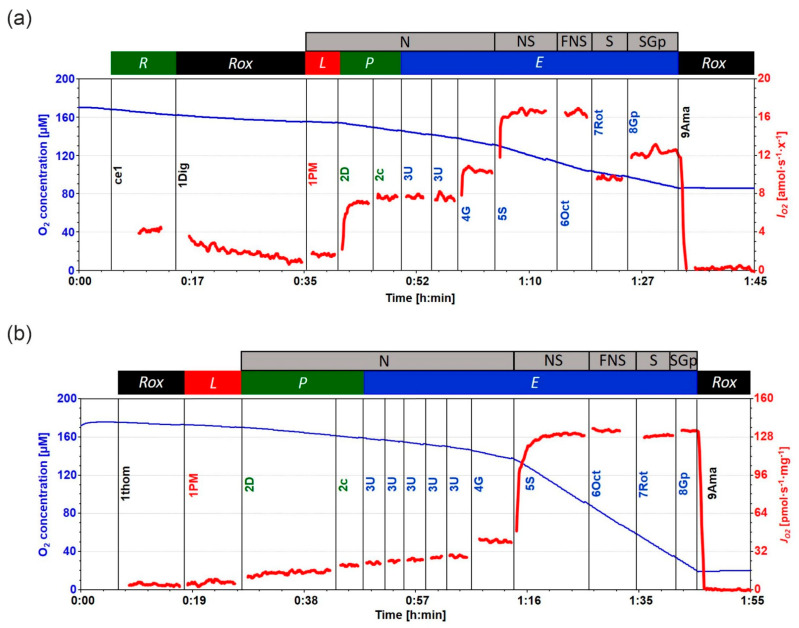
Representative traces of substrate-uncoupler-inhibitor titration (SUIT, SUIT-001 O2 ce-pce D003, and SUIT-001 O2 mt D001) protocol for measurement of (**a**) PBMC and (**b**) liver mitochondrial respiration using high-resolution respirometry. (**a**) **ce1**: ROUTINE respiration (*R*) was measured in the presence of isolated living PBMCs. **1Dig** (2 µg/mL): Plasma membrane was permeabilized using digitonin, inducing residual oxygen consumption *Rox*. **1PM**: nonphosphorylating LEAK respiration (*L*) was assessed by injecting pyruvate (P, 5 mM) and malate (M, 2 mM) as N-linked substrates in the absence of adenylates, PM*_L_*. **2D** (2.5 mM ADP): OXPHOS capacity (*P*) was measured by adding ADP at kinetically saturating concentration; PM*_P_*. **2c** (10 µM cytochrome *c*): cytochrome *c* was added to test for the mitochondrial outer membrane integrity, PM*_cP_*. **3U** (0.5 µM steps of CCCP): stepwise titrations of the protonophore carbonyl cyanide m-chloro phenyl hydrazine (CCCP) to measure electron transfer (ET) capacity (*E*), PM*_E_*. **4G** (10 mM glutamate): glutamate was added to measure N-linked ET capacity, PGM*_E_*. **5S** (10 mM succinate): addition of succinate for the simultaneous action of N-linked substrates and succinate with convergent electron flow in the NS-pathway for reconstitution of the TCA cycle function, PGMS*_E_*. **6Oct** (0.5 mM Oct): titration of octanoylcarnitine enabled the simultaneous action of F- and N-linked substrates and S with convergent electron flow in the FNS pathway for reconstitution of TCA cycle function, and additive or inhibitory effect of F-linked substrate Oct to support fatty acid oxidation, OctPGMS*_E_*. **7Rot** (0.5 µM rotenone): CI inhibition by rotenone-induced succinate-linked ET capacity, S*_E_*. **8Gp** (10 mM Gp): titration of glycerol-3-phosphate provided simultaneous action of convergent S- and Gp-linked electron entry in the SGp-pathway, SGp*_E_*. **9Ama** (2.5 µM Ama): injection of antimycin A blocked CIII and induced the state of residual oxygen consumption, *Rox*. Experiment: 2020-07-22 PS3-01 IDA 6 Chamber A. O_2_ concentration (µM) (blue trace) and O_2_ flow per cell *I_O2_* (amol·s^−1^·cell^−1^) (red trace). (**b**) **1thom**: *Rox* measured in the presence of liver homogenate, *Rox*; for titrations 1PM, 2D, 2c, 3U, 4G, 5S, 6Oct, 7Rot, 8Gp, and 9Ama, see legend (**a**). Experiment: 2020-07-22 PS3-01 IDA 6 Chamber B. O_2_ concentration (µM) (blue trace) and mass-specific flux (pmol·s^−1^·mg^−1^) (red trace).

We then analyzed the effects of IDA on mitochondrial respiratory capacity in freshly isolated PBMCs and homogenate from fresh liver biopsies (for protocols, see Figure 6a,b). Of note, we observed an increase of the mitochondrial respiration in the PBMCs of the IDA compared to the control rats (Figure 2a). This included LEAK respiration (Figure 2a, state PM*_L_*), OXPHOS capacity (Figure 2a, state PM*_P_*), and electron transfer (ET) capacity (Figure 2a, states PM*_E_*, PGMS*_E_*, and S*_E_*). In contrast, no major changes in liver mitochondrial respiratory capacity were observed when comparing IDA and control rats (Figure 2b).

**Figure 3:**
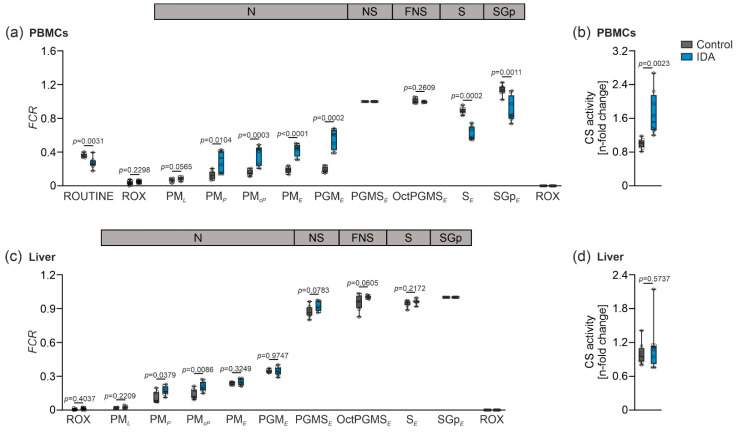
Mitochondrial respiration in PBMCs and liver from control and IDA rats independent of mitochondrial density and citrate synthase (CS) activity. (**a**) *FCR* calculated from PBMC respiration with reference state PGMS*_E_*. States: ROUTINE: living cells; ROX: digitonin; PM*_L_*: PM; PM*_P_*: ADP; PM*_cP_*: *c*; PM*_E_*: uncoupler; PGM*_E_*: G; PGMS*_E_*: S; OctPGMS*_E_*: Oct; S*_E_*: rotenone; SGp*_E_*: Gp; ROX: antimycin A. (**c**) *FCR* calculated from liver respiration with reference state SGp*_E_*. States: ROX: liver homogenate; PM*_L_*: PM; PM*_P_*: ADP; PM*_cP_*: *c*; PM*_E_*: uncoupler; PGM*_E_*: G; PGMS*_E_*: S; OctPGMS*_E_*: Oct; S*_E_*: rotenone; SGp*_E_*: Gp; ROX: antimycin A. (**b**,**d**) CS activity in PBMCs and liver, respectively. Values of CS activity are shown as n-fold change of control. *N* = 8 rats per group. An unpaired two-tailed Student’s *t*-test was applied for all normal distributed results; a Mann-Whitney U-test was applied for the nonparametric results (**a**) PM*_P_*, PM*_cP_*, PGM*_E_*, S*_E_*; (**b**) PM*_P_*, and (**d**). Values are shown as median ± interquartile range. *p*-Values are shown in the graphs.

We next investigated whether the tissue-specific alterations of the mitochondrial function could be based on changes in the mitochondrial quality and density in response to IDA. Therefore, flux control ratios (*FCR*) were calculated in both PBMCs and the liver, whereby the values obtained from the measurements of the mitochondrial respiration were normalized for a common reference state (PBMCs: PGMS*_E_*; liver: SGp*_E_*) showing mitochondrial respiration independent of mitochondrial density (Figure 3a,c) [26,27]. In PBMCs, increased mitochondrial respiration was still observed after normalization independent of mitochondrial density in OXPHOS, indicating a change in mitochondrial quality (Figure 3a, state PM*_P_*) and ET (Figure 3a, states PM*_E_* and S*_E_*). Furthermore, citrate synthase (CS) activity, a marker for mitochondrial density, was measured [28,29,30]. In PBMCs, CS activity was significantly increased (Figure 3b). Consequently, the observed changes in the mitochondrial respiration in PBMCs originated from alterations in both mitochondrial quality and mitochondrial density. In the liver, we found a slight increase in the OXPHOS capacity, whereas all other measurements of mitochondrial function remained unaffected when comparing the control to the IDA animals (Figure 3c, state PM*_P_*). CS activity in the liver did not show any differences (Figure 3d), indicating that the mitochondrial density remained unaltered when comparing the IDA with the control rats, and supporting the conclusion of the unchanged mitochondrial quality drawn from the *FCR*.

**Figure 4:**
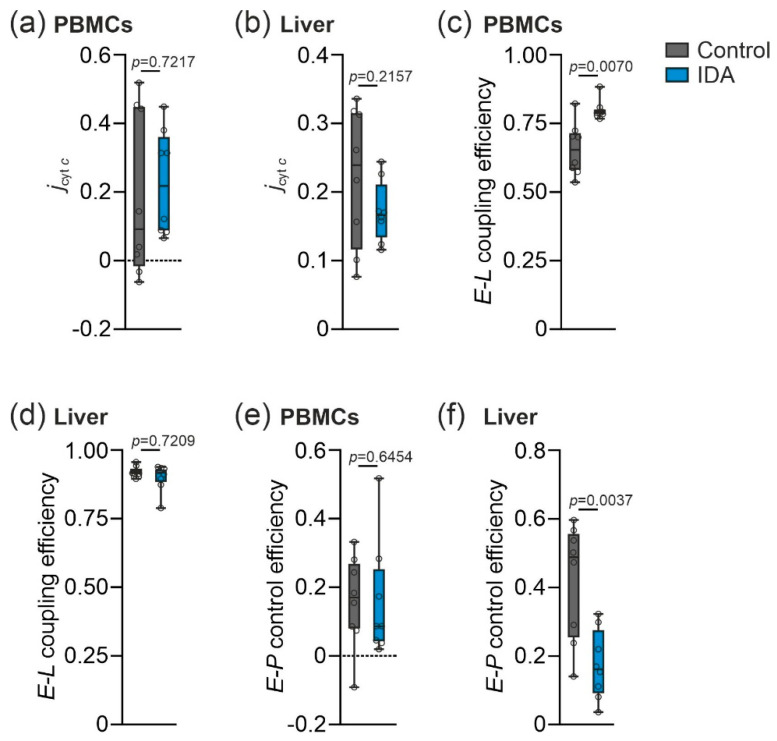
Effects of IDA on flux control efficiencies in rat PBMCs and liver. (**a**,**b**) Cytochrome *c* control efficiency (*j*_cyt *c*_) indicates integrity of the outer mitochondrial membrane. (**c**,**d**) *E-L* coupling efficiency (*j_E-L_*) indicates preserved coupling of electron transfer to phosphorylation of ADP. (**e**,**f**) *E-P* control efficiency indicates the limitation of the OXPHOS capacity due to the capacity of the phosphorylation system. *N* = 8 rats per group. An unpaired two-tailed Student’s *t*-test was applied for all normal distributed results; a Mann-Whitney U-test was applied for the nonparametric results shown in (**c**–**e**). Values are shown as median ± interquartile range. *p*-Values are shown in the graphs.

Having observed the tissue-specific differences in the mitochondrial function in PBMCs compared to the liver of the control and IDA animals, we next studied for the possibility of mitochondrial damage. As a surrogate, we investigated a potential loss of the mitochondrial outer membrane (mtOM) integrity by quantifying the cytochrome *c* control efficiency (*j*_cyt *c*_) [27]. The *j*_cyt *c*_ did not show significant differences either in PBMCs or in the liver when comparing the control to the IDA rats (Figure 4a,b). Consequently, the damage of the mtOM could be ruled out as a cause for the differences in PBMC mitochondrial respiration between the two groups. Furthermore, in the PBMCs of the IDA rats, an increase in the *E-L* coupling efficiency was detected (Figure 4c), indicating an improved coupling of the ET to the phosphorylation of ADP. This may likewise be underlying the observed increase in mitochondrial respiration. In the liver, the *E-L* coupling efficiency remained stable (Figure 4d), whereas the *E-P* control efficiency was decreased in the IDA liver homogenates as compared to the controls (Figure 4f), indicating a decreased capacity of the oxidative phosphorylation system [27,31]. In PBMCs, the *E-P* control efficiency remained unchanged (Figure 4e).

### 2.3. PBMC Mitochondrial Respiration was Not Correlated with Liver Mitochondrial Respiration

**Figure 5:**
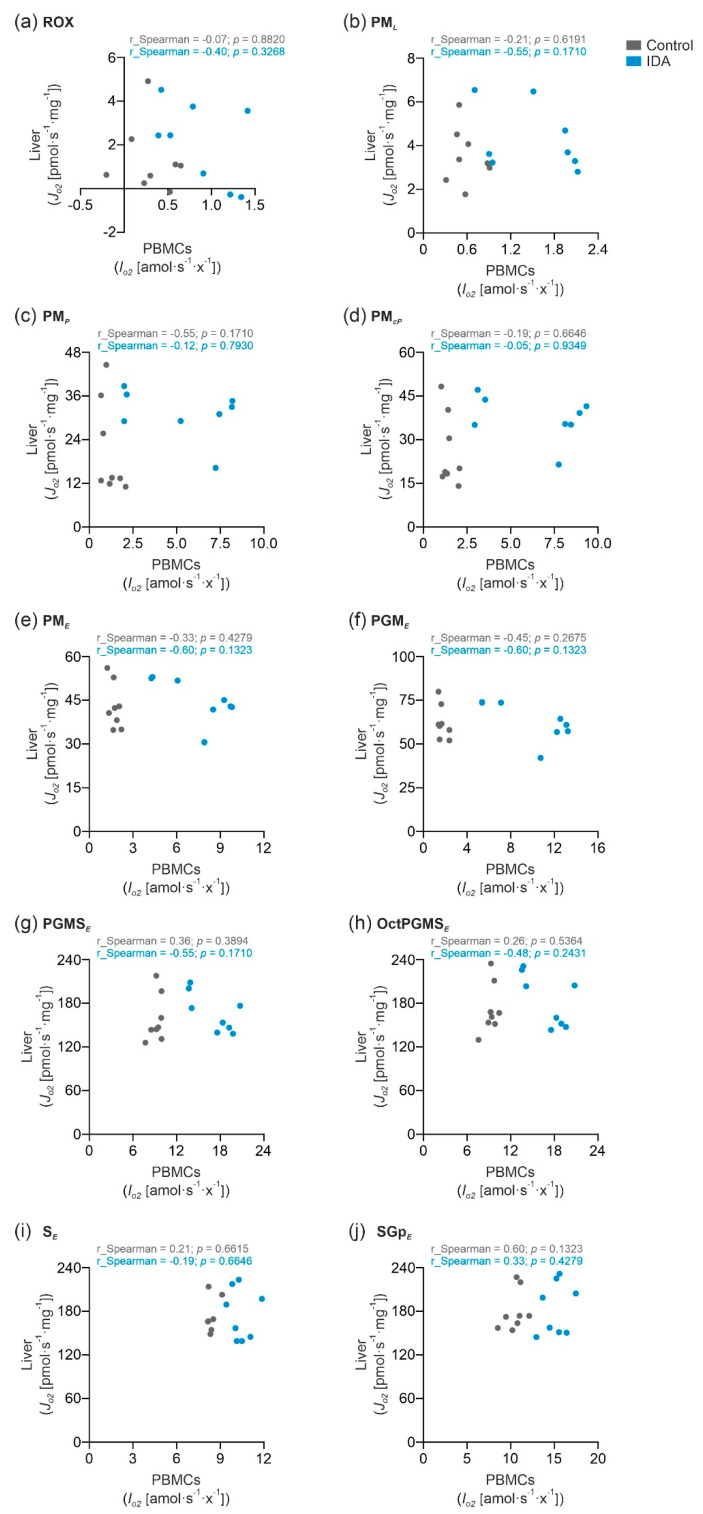
Correlation analysis of PBMC mitochondrial respiration and liver mitochondrial respiration in control and IDA. Spearman’s rank correlation in the measured respiratory states in PBMCs and liver: (**a**) ROX; (**b**) PM*_L_*; (**c**) PM*_P_*; (**d**) PM*_cP_*; (**e**) PM*_E_*; (**f**) PGM*_E_*; (**g**) PGMS*_E_*; (**h**) OctPGMS*_E_*; (**i**) S*_E_*; (**j**) SGp*_E_*. *N* = 8 rats per group. Statistical significance of the correlation was tested using an unpaired two-tailed Student’s *t*-test. The correlation coefficients (*r*_Spearman) and the *p*-values are shown in the graphs.

To investigate if the mitochondrial respiration in PBMCs reflects the liver mitochondrial respiration, we analyzed the correlation of the respiratory states shown in Section 2.2. (Figure 5a–j). In none of the states was the PBMC mitochondrial respiration significantly linked to the liver mitochondrial respiration. Consequently, the measurement of the mitochondrial respiration in the PBMCs does not reflect the liver mitochondrial function. Nevertheless, PBMCs may be used as a surrogate for mitochondrial dysfunction in specific diseases affecting other organs or tissues.

## 3. Discussion

We found that IDA led to different effects on the mitochondrial respiratory capacity in permeabilized PBMCs and liver homogenates. Surprisingly, IDA resulted in an increase of mitochondrial activity in PBMCs, for which different factors may be responsible. We observed a relative expansion of monocytes when comparing PBMCs from the IDA versus the control animals. Thus, it will be of interest in future investigations to study if the mitochondrial respiratory capacity differs between the various leucocyte species in general, but also in response to iron deficiency, as suggested in previous studies [32,33]. However, in contrast to these studies, we did not detect any differences in the mitochondrial outer membrane integrity as determined by the *j*_cyt *c*_, but we found an increase in the mitochondrial density in PBMCs, as shown by the higher CS activity, likewise explaining the increase in the mitochondrial respiration. Nonetheless, the increase in the mitochondrial activity in the PBMCs of the IDA rats was surprising because iron deficiency in vitro has been demonstrated to result in impaired Fe-S cluster synthesis for the respiratory complexes and in reduced mitochondrial activity [10,11,34]. However, most of those studies have been performed in vitro and iron deficiency was often induced by the addition of an iron chelator. Nonetheless, the increase in the mitochondrial density and changes in the quality suggests the presence of a compensatory mechanism, by which the mitochondrial number or the longevity of mitochondria and the quality of mitochondria compensate for the impaired mitochondrial activity as a consequence of the iron deficiency. This is in line with a recent observation indicating that the age of mitochondria determines their metabolic activity [35]. Moreover, in future studies, mitochondrial morphology could be investigated in PBMCs and the livers of the control and IDA rats to gain further knowledge about the underlying causes of the differences in the mitochondrial respiration observed in the present study.

Compared to the results of the mitochondrial activity in cardiomyocytes and the skeletal muscle fibers described in the literature, the mitochondria in the liver did not show a decrease in the mitochondrial respiration induced by IDA in our study [14,15,16,17,18]. Plausible explanations might be that those studies on the morphological changes of the mitochondria in different organs and cells used mostly animals whose iron deficiency or IDA was induced over a longer period of time [14,15,16,17,18,19,20]. In contrast, we used a short-term phlebotomy model to induce IDA that might not reduce the amount of iron in the liver to an extent required for restricting the iron supply to the mitochondria, leading to impairment of the mitochondrial respiration. In addition, the liver is a central organ of iron storage and iron regulation. Therefore, short-term induction of iron deficiency by phlebotomy may be compensated by iron mobilization within this organ, thereby delivering enough iron to maintain hepatic mitochondrial function. It will be of interest to study if long-lasting persistence of iron deficiency changes the phenotype of the hepatic mitochondrial function. This is indicated by observations made in mice with genetic and dietary iron overload, where only long-term exposure to those stressors resulted in altered mitochondrial iron status and activity [36]. Finally, it has to be kept in mind that iron availability affects multiple other metabolic pathways, including tricarboxylic acid (TCA) cycle activity, lipid, and protein synthesis, which may affect the mitochondrial structure and function [37].

Our study suggests that the mitochondrial function in PBMCs and the liver is not comparable, at least in the setting of IDA. Therefore, changes in mitochondrial activity in PBMCs are not associated with alterations of the mitochondrial function in the liver. Our results do not exclude, however, that mitochondrial activity in PBMCs may indicate principal defects or alterations of mitochondrial function present in specific diseases affecting other organs or tissues.

## 4. Materials and Methods

### 4.1. Animal Experiments

All rat experiments were performed as described before [25]. Age-matched female Lewis rats (Charles River Laboratories, Margate, UK) were kept on a standard rodent diet containing 180 mg Fe/kg (Ssniff, Soest, Germany) until they reached an age of 8 to 11 weeks. The animals had free access to food and water and were kept according to institutional and governmental guidelines in the animal housing unit of the Medical University of Innsbruck, with a 12 h light-dark cycle and an average temperature of 20 ± 1 °C. All animal experiments were approved by the Austrian Federal Ministry of Science and Research (BMWFW-66.011/0138-WF/V/3b/2016).

Half of the rats were phlebotomized; 1.8 mL blood was sampled daily for five consecutive days (starting one week before death) to induce IDA. After termination of the experiment, organs and blood were harvested for further analysis. Total blood counts were measured using a VetABC animal blood counter (Scil Animal Care, Viernheim, Germany).

### 4.2. Reticulocyte Quantification

Peripheral reticulocytes were measured using flow cytometry. Briefly, full blood was stained with thiazole orange (Santa Cruz, Dallas, TX, USA), and after identification of the single cells using forward and side scatters, the thiazole-orange-positive cells were identified as reticulocytes.

### 4.3. Isolation of PBMCs

PBMCs were isolated as described before [21]. Briefly, rat full blood was diluted 1:3 with phosphate-buffered saline (PBS, Lonza Bioscience, Basel, Switzerland) and loaded onto Pancoll separating solution (density 1.077 g/mL, PAN-Biotech, Aidenbach, Germany). After centrifugation (1000 *g*, 10 min, no brake), the buffy coat was collected and the cells were washed twice with PBS.

### 4.4. High-Resolution Respirometry

All measurements were performed as described before [26,27,36]. Briefly, mitochondrial respiration was performed using the Oxygraph-2k (O2k, Oroboros Instruments, Innsbruck, Austria). Fresh rat liver tissue samples were collected and homogenized, and PBMCs were isolated from blood samples. Respiratory measurements of isolated living PBMCs (cell concentration 2 × 10^6^ x/mL) and liver tissue homogenate (0.5 mg/mL) were performed at kinetically saturating oxygen concentrations in mitochondrial respiration medium MiR05-Kit (Oroboros Instruments, Innsbruck, Austria) containing 0.5 mM ethylene glycol tetraacetic acid (EGTA), 3 mM magnesium chloride (MgCl_2_), 60 mM lactobionic acid, 20 mM taurine, 10 mM monopotassium phosphate (KH_2_PO_4_), 20 mM 4-(2-hydroxyethyl)-1-piperazineethanesulfonic acid (HEPES), and 110 mM D-sucrose supplemented with 1 g/L essentially fatty acid-free bovine serum albumin (BSA, Sigma-Aldrich, St. Louis, MO, USA). Manual titrations of substrates, uncoupler, and inhibitors were performed using Hamilton syringes (customized for Oroboros Instruments, Hamilton Central Europe, Giarmata, Romania). Figure 6a,b shows the substrate-uncoupler-inhibitor titration (SUIT, SUIT-001 O2 ce-pce D003, and SUIT-001 O2 mt D001) protocol used in the experiments for isolated living PBMCs and liver homogenate, respectively [38,39]. Data analysis was performed using the software DatLab 7.4 (Oroboros Instruments). Flux control efficiencies were calculated according to the following definitions [8]: Cytochrome *c* control efficiency (*j*_cyt *c*_ = 1 − PM*_P_*/PM*_cP_*); *E-L* coupling efficiency (*j_E-L_* = 1 − PM*_L_*/PM*_E_*); *E-P* control efficiency (1 − PM*_cP_*/PM*_E_*).

### 4.5. Plasma and Total Tissue Iron

Plasma iron was measured using QuantiChrom Iron Assay kit (BioAssay Systems, Hayward, CA, USA) according to the manufacturer’s instructions. Tissue iron determination was performed as described [27,36,40]. After acidic hydrolysis at 65 °C for 24 h, the iron content was measured using a colorimetric staining solution containing sodium acetate and bathophenanthroline disulfonic acid. Total tissue iron content was normalized by protein content.

### 4.6. RNA Extraction and Quantitative Real-Time PCR

Liver total RNA was extracted using TRI reagent (Sigma-Aldrich) according to the manufacturer’s protocol. After reverse transcription, mRNA expression was analyzed as described [36,41]. The following primers were used: *Hamp* forward 5′-TGAGCAGCGGTGCCTATCT-3′, *Hamp* reverse 5′-CCATGCCAAGGCTGCAG-3′, *Hamp* probe FAM-CGGCAACAGACGAGACAGACTACGGC-BHQ1, *Gusβ* forward 5′-ATTACTCGAACAATCGGTTGCA-3′, *Gusβ* reverse 5′-GACCGGCATGTCCAAGGTT-3′, and *Gusβ* probe FAM-CGTAGCGGCTGCCGGTACCACT-BHQ1. Quantitative real-time PCR reactions were performed on the CFX96 PCR system (BioRad, Hercules, CA, USA). Relative gene expression was calculated with the ΔΔCt method in the CFX96 Manager software (BioRad). The housekeeping gene *Gusβ* was used as reference control.

### 4.7. CS Activity

CS activity was measured as described [27,36]. A spectrophotometric assay was used to measure the enzyme activity in snap-frozen liver homogenates. The sample enzymatic reaction mix contained 0.25% Triton X-100 in aqua dest, 0.31 mM acetyl-coenzyme A in aqua dest, 0.1 mM 5,50-dithiobis-(2-nitrobenzoic acid) in 1 M Tris-HCl buffer (pH 8.1), and 0.5 mM oxaloacetate in 0.1M triethanolamine-HCl buffer (pH 8.0). The absorbance of the reaction product, thionitrobenzoic acid, was measured at 412 nm over 200 s. The resulting enzyme activities were normalized by the protein content of the samples.

### 4.8. Statistics

Statistical analysis was carried out using the software GraphPad Prism 9. In case of normal distribution, statistical significance was determined by an unpaired two-tailed Student’s *t*-test. Otherwise, a Mann-Whitney U-test was applied. To assess whether PBMC mitochondrial respiration was correlated to liver mitochondrial respiration, a Spearman’s rank correlation analysis with a two-tailed Student’s test was performed. Data was shown as median ± interquartile range. *p*-Values below 0.05 were considered significant.

## Data Availability

All data presented within this study is available within the manuscript.
